# Coloring Activities for Anxiety Reduction and Mood Improvement in Taiwanese Community-Dwelling Older Adults: A Randomized Controlled Study

**DOI:** 10.1155/2020/6964737

**Published:** 2020-01-21

**Authors:** Malcolm Koo, Hsuan-Pin Chen, Yueh-Chiao Yeh

**Affiliations:** ^1^Graduate Institute of Long-term Care, Tzu Chi University of Science and Technology, Hualien City, Taiwan; ^2^Dalla Lana School of Public Health, University of Toronto, Toronto, Ontario, Canada; ^3^Master's Program in Natural Healing Sciences, Nanhua University, Chiayi, Taiwan; ^4^Department of Natural Biotechnology, Nanhua University, Chiayi, Taiwan

## Abstract

To investigate the effect of mandala coloring, plaid pattern coloring, and free-form drawing activities on anxiety and mood in older Taiwanese adults. A total of 120 older adults aged 55 years to 75 years were recruited from 18 community-based learning centers for older adults in southern Taiwan. They were randomly assigned to engage in one of the following four activities for 20 minutes: (1) mandala coloring group, (2) plaid pattern coloring group, (3) free-form drawing group, and (4) reading group (control). Information on sociodemographic, lifestyle, and perceived health status was collected at the baseline. In addition, anxiety levels, measured using the 20-item State-Trait Anxiety Inventory–State Anxiety Scale (STAI-S), were ascertained at the baseline (T1), after a brief anxiety induction (T2), and at the end of the assigned activity (T3). The mean anxiety levels among the four groups at T3 were analyzed using analysis of covariance, followed by Šidák multiple comparison test, as appropriate. The mean age of the 120 study participants was 65.1 years and 73.3% were females. A significantly lower anxiety level was observed only in the mandala coloring group (least square mean = 28.2; 95% confidence interval = 24.7–31.7) compared with the control group (least square mean = 36.0; 95% confidence interval = 32.9–39.2) (*P*=0.004, partial eta-squared = 0.113). Furthermore, when the STAI-S was analyzed at the item level, the mandala coloring group was significantly different from the control group in the following six feelings: calmed down, safe, at ease, rested, satisfied, and I feel good. In conclusion, short-term mandala coloring activity could significantly alleviate self-induced anxiety in community-dwelling older adults. Further studies on the long-term effects of mandala coloring activity in improving the emotional well-being of older adults are warranted.

## 1. Introduction

With a global trend in population aging, the issue of anxiety has become a widespread health concern [1]. Epidemiological studies have estimated that the global prevalence of anxiety disorders in older adults ranges from 0.9% to 28.3%, and they are highly associated with social, physical, psychological, and economic impacts [2]. The discomfort caused by late-life anxiety can adversely impact on the physical and psychological health status, which may lead to depression and increased health care costs [1].

Various psychosocial interventions with participatory arts have been explored to alleviate anxiety, depression, and negative mood in older adults [3–6]. A number of studies have suggested that coloring a mandala could serve as an effective intervention for alleviating anxiety and improving mood [7–11]. A mandala is a spiritual geometric configuration of symbols used in various religions. In modern use, it usually means a geometric pattern, typically with a radial balance that is associated with a sense of wholeness and healing [12]. The protocol used in the present study was similar to two previous studies [7, 8]. In the earlier study by Curry and Kasser [7], the study participants were 84 undergraduate students, whereas the replication study by van der Vennet and Serice [8] investigated 50 graduate and undergraduate students. However, to the best of our knowledge, no studies have been conducted on community-dwelling older adults in Taiwan. Therefore, the aim of this randomized controlled trial was to compare the short-term effect of mandala coloring, plaid pattern coloring, and free-form color drawing in alleviating self-induced anxiety in older adults recruited from community-based learning centers for older adults in southern Taiwan.

## 2. Materials and Methods

### 2.1. Setting and Study Population

The protocol of this study was approved by the Human Research Ethics Committee of National Chung Cheng University (No. CCUREC105071801). All study participants provided a written informed consent form prior to the beginning of the intervention.

Study participants aged 55 to 75 years were recruited using convenient sampling from 18 community-based learning centers for older adults in southern Taiwan. A poster was put up on the notice board in each center to describe the study and to recruit potential participants. Participants who were using medications for anxiety, depression, or severe mental disorder were excluded from this study.

The sample size was determined by G∗Power software (version 3.1.9.4) with a Cohen's f effect size estimated to be 0.31, based on means and standard deviations reported by van der Vennet and Serice [8], with a power of 0.80 and an alpha of 0.05. An estimated sample size of 30 participants per group would be required.

### 2.2. Experimental Design and Outcome Assessment

Participants were randomly assigned to engage in one of the following four activities for 20 minutes: (1) mandala coloring, (2) plaid pattern coloring, (3) free-form drawing, and (4) reading (control group). A sequence of 1 to 4, generated using the random number generator in Microsoft Excel software, was prepared in advance and placed in opaque, sealed envelopes. The envelopes were opened sequentially for each consented participant to determine the allocation sequence. Information on sociodemographic, lifestyle, and perceived health status was collected at the baseline. In addition, the level of anxiety, measured using the 20-item Spielberger State-Trait Anxiety Inventory–State Anxiety Scale (STAI-S) [13], was ascertained at the baseline (T1), after a brief anxiety induction (T2), and at the end of the assigned activity (T3). To induce an anxious emotional state in the study participants, they were asked to recall a prior event in their lives that they felt most fearful and then to write down their experiences on a piece of unlined A4-sized paper (21.0 cm × 29.7 cm). This brief induction period lasted for approximately four minutes [7, 8]. The intervention was conducted on each participant individually at the community-based learning centers.

All participants except those in the control group were provided with six colored pencils (red, orange, yellow, green, blue, and purple) and were given 20 minutes for completing their coloring or drawing task. Participants in the mandala coloring and plaid pattern coloring groups were given an outline of a mandala or irregular plaid pattern, respectively, printed on an A4-sized paper. Participants in the free-form drawing group were given a blank piece of A4-sized paper. The design of the mandala and the plaid pattern were identical to those used in the study by Curry and Kasser [7]. Both designs were composed of 324 areas of various shapes and sizes.

The main outcome measure of this study was anxiety levels, which were measured using the STAI-S. The STAI contains two subsets of items: (1) state anxiety (STAI-S), which is designed to assess an individual's reaction to stress and emotional state at a particular time, and (2) trait anxiety (STAI-T), which is related to an individual's personality traits and stress perceptions [13]. Participants were asked to rate their current mood states toward each of the items in the inventory on a 4-point Likert scale. For the purpose of the present study, only the 20-item STAI-S was used. The total score of STAI-S ranges from 20 to 80, with higher scores suggesting higher perceived anxiety levels. The Chinese version of the STAI, translated and revised by Chung and Long (1984) with good internal consistency reliability (Cronbach's *α* = 0.93), was used for this study [14].

### 2.3. Statistical Analysis

Continuous variables are expressed as mean and standard deviation. Categorical variables are expressed as number and percentage. Chi-square test or analysis of variance was used to compare group differences in the baseline characteristics of the participants, as appropriate. Analysis of covariance (ANCOVA) of postintervention (T3) anxiety levels was conducted to adjust for postinduction (T2) anxiety levels, sex, and educational levels. Šidák post hoc test was used to adjust for multiple pairwise comparisons when overall ANCOVA was significant [15]. All analyses were performed with IBM SPSS Statistics for Windows, Version 24.0 (IBM Corp., Armonk, NY, USA). A two-tailed *P* value of less than 0.05 was considered significant.

## 3. Results

**Figure 1:**
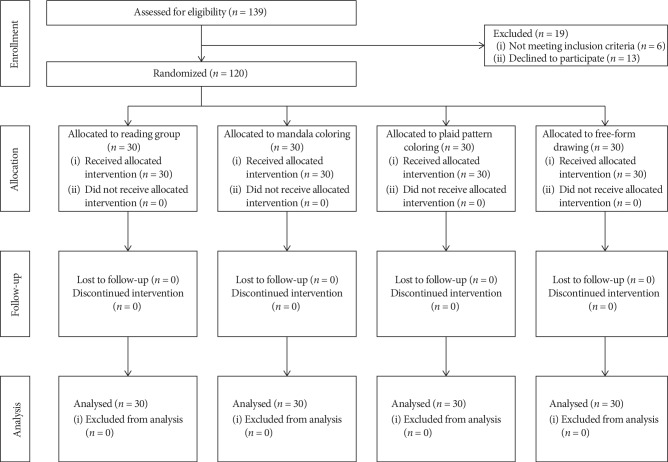
CONSORT flow chart for the study.

A total of 139 older adults were assessed for eligibility, and 13 declined to participate.  Figure 1 presents a CONSORT flow chart. The mean age of the 120 participants was 65.1 years (standard deviation = 5.7 years), and 73.3% were females. The baseline characteristics between the four groups were similar except for sex (*P*=0.049) and educational level (*P*=0.040) (Table 1). These two variables were included in the model during subsequent ANCOVA. No adverse events were noted in any participants during the study period. Comparisons of the total scores of STAI-S at the three different time points (T1, T2, and T3) are shown in Table 2. No significant differences between the groups were observed at T1 (*P*=0.346) and T2 (*P*=0.457), indicating that the randomization procedure was successful.

Table 3 shows the least square mean scores of the STAI-S for the four groups at the postanxiety induction (T2) and at the postintervention (T3). No significant differences were observed at T2, indicating that anxiety was similarly induced among the four groups. Comparisons of least square mean scores of STAI-S at T3, the main outcome of this study, showed that the anxiety level of the mandala coloring group (least square mean = 28.2; 95% confidence interval = 24.7–31.7) was significantly lower than that of the control group (least square mean = 36.0; 95% confidence interval = 32.9–39.2) (*P*=0.004). A partial eta-squared of 0.113 was found indicating that the effect size was above medium. A partial eta-squared of 0.06 and 0.14 could be considered as to represent a medium and large effect size, respectively [16].

Furthermore, when the STAI-S was analyzed at the item level, only the mandala coloring group was significantly different from the control group in the following six feelings: calmed down, safe, at ease, rested, satisfied, and I feel good. None of the items in the plaid pattern coloring group or free-form drawing group were significantly different from the control group, except the item “satisfied” in the free-form drawing group (Table 4).

## 4. Discussion

The present randomized controlled trial showed that a single 20-minute session of mandala coloring activity could significantly alleviate self-induced anxiety in community-dwelling older adults. The effect size was found to be above medium. In addition, a number of positive emotions, including calmed down, safe, at ease, rested, satisfied, and I feel good, were significantly improved with mandala coloring activity. On the other hand, none of the item scores was significantly different between the plaid pattern coloring group and the control group, suggesting that the pattern, rather than the coloring activity *per se* contributes to an anxiety-lowering effect. Regarding the free-form drawing group, only the least square mean of the item “satisfied” was significantly lower than that of the control, which again suggests that the geometric pattern of the mandala instead of the drawing activity *per se* could lead to an anxiety-lowering effect.

In the randomized controlled trial of 84 undergraduate students, Curry and Kasser [7] reported that anxiety levels were significantly declined in the mandala coloring and plaid pattern coloring groups, compared with free-form drawing group. The authors hypothesized that the complexity and structure of the mandala and plaid design could engage participants into a meditative-like state that help to reduce anxiety. However, in the present study on community-dwelling older adults, we found that only mandala coloring could lead to a significant decrease in anxiety levels. This difference could be attributed to a difference in age and cultural background in our participants. In addition, a replication study [8] of that by Curry and Kasser [7] showed that coloring a mandala (*P*=0.05), but not a plaid design (*P*=0.23) when compared with free-form coloring, could reduce the anxiety levels in 50 graduate and undergraduate students. In addition, the mean anxiety level in the mandala coloring group was also significantly different from that of the plaid pattern coloring group (*P*=0.02). The authors concluded that while both mandala and plaid pattern were similarly complex, the circular design of the mandala might be inherently meditative. The word “mandala” means the “whole world” or “healing circle,” and it is used to describe circular designs used in certain cultures for mediation [17]. Our study also showed that the anxiety-lowering effect was significantly greater in the mandala coloring group compared with any of the other three groups. Results from these two studies [7,8] and our study appear to be congruent with the Jungian school of thought that the mandala had calming and healing effect upon its maker or viewer [18].

When the STAI-S was further analyzed at the item level, we found that the mandala coloring group showed a significantly lower score compared with the control group in six positive emotions, including calmed down, safe, at ease, rested, satisfied, and I feel good. Conversely, mandala coloring was unable to significantly improve negative emotions compared with the controls. This is also in line with Jung's description of his patients' experience as calming, positive, and soothing when creating a mandala [18].

The duration of the intervention was 20 minutes in the present study. Whether the duration could influence the effect of free-form drawing and plaid pattern coloring will require further study. Moreover, the optimal duration for the mandala coloring will need additional investigation. A single group quasiexperimental study on 100 university students showed that a 30-minute mandala coloring was able to significantly reduce the level of anxiety, as measured by the STAI [11]. The studies conducted by Curry and Kasser [7] and van der Vennet and Serice [8] also allowed participants to color for 20 minutes. However, a recent randomized controlled trial of mandala coloring using an iPad on acute pain symptoms in pediatric patients reported a significantly reduced heart rate and anxiety experienced by patients with only 5 minutes of mandala intervention [19]. With the use of a 12-minute session, another randomized controlled trial revealed that coloring a blank piece of paper or a mandala were equally effective in reducing anxiety in students [12]. According to aforementioned studies, it is clear that additional studies are needed to establish whether there exists a minimum or optimal duration of mandala coloring to maximize its effectiveness for anxiety reduction.

There were several limitations to this study that should be addressed in future research. First, only one mandala design, identical to that used in the studies by Curry and Kasser [7] and van der Vennet and Serice [8], was used in the present study. Whether different geometric designs could yield different results will require further investigations. In addition, whether there exist special elements or their combinations, such as the spatial arrangement of circles, squares, semicircles, triangles, and the numbers of 8 and 4, that must be present for a mandala to be effective in alleviating anxiety will need to be explored [20]. Second, in this study, acute anxiety was self-induced by asking the participants to recall a prior fearful event in their lives. Whether mandala coloring could effectively manage chronic anxiety will require longitudinal observational studies to clarify. Third, given the nature of the intervention, neither the participants nor the research assistant was masked of the allocation of the intervention. Nevertheless, the data analyst was unaware of the group allocation when analyzing the data.

## 5. Conclusion

In this randomized controlled trial of community-dwelling older adults, engaging in a 20-minute mandala coloring activity could significantly alleviate self-induced anxiety. Further studies on the long-term effect of mandala coloring activity in improving the emotional well-being of older adults are warranted.

## Figures and Tables

**Table 1:** Baseline characteristics of the study participants (*N* = 120).

Variable	*N* (%) unless otherwise stated					*P*
	Total (*N* = 120)	Control (*N* = 30)	Mandala coloring (*N* = 30)	Plaid pattern coloring (*N* = 30)	Free-form drawing (*N* = 30)	
Age (year), mean (SD)	65.1 (5.7)	64.7 (5.3)	65.4 (6.4)	66.1 (5.2)	64.4 (5.8)	0.672

Body mass index (kg/m^2^), mean (SD)	23.4 (2.9)	23.2 (3.3)	23.3 (2.5)	23.3 (3.1)	23.7 (2.8)	0.909

Sex						0.049
Male	32 (26.7)	9 (30.0)	4 (13.3)	13 (43.3)	6 (20.0)	
Female	88 (73.3)	21 (70.0)	26 (86.7)	17 (56.7)	24 (80.0)	

Educational level						0.040
Elementary school or below	24 (20.0)	3 (10.0)	4 (13.3)	7 (23.3)	10 (33.4)	
Middle school	10 (8.3)	3 (10.0)	4 (13.3)	3 (10.0)	0 (0.0)	
High school	52 (43.3)	9 (30.0)	14 (46.7)	16 (53.4)	13 (43.3)	
College or above	34 (28.4)	15 (50.0)	8 (26.7)	4 (13.3)	7 (23.3)	

Marital status						0.138
Single	8 (6.7)	3 (10.0)	2 (6.6)	0 (0.0)	3 (10.0)	
Married	81 (67.5)	23 (76.7)	20 (66.7)	17 (56.7)	21 (70.0)	
Divorced/widowed	31 (25.8)	4 (13.3)	8 (26.7)	13 (43.3)	6 (20.0)	

Religious affiliation						0.525
No	29 (24.2)	7 (23.3)	6 (20.0)	8 (26.7)	8 (26.7)	
Buddhism	66 (55.0)	19 (63.4)	18 (60.0)	12 (40.0)	17 (56.7)	
Taoism	23 (19.2)	4 (13.3)	6 (20.0)	9 (30.0)	4 (13.3)	
Christian/catholicism/other	2 (1.6)	0 (0.0)	0 (0.0)	1 (3.3)	1 (3.3)	

Exercise						0.612
No	5 (4.2)	1 (3.3)	1 (3.3)	2 (6.7)	1 (3.3)	
Irregular	57 (47.5)	11 (36.7)	13 (43.3)	15 (50.0)	18 (60.0)	
Regular	58 (48.3)	18 (60.0)	16 (53.4)	13 (43.3)	11 (36.7)	

Smoking habit						0.388
No	119 (99.2)	30 (100.0)	29 (96.7)	30 (100.0)	30 (100.0)	
Yes	1 (0.8)	0 (0.0)	1 (3.3)	0 (0.0)	0 (0.0)	

Alcohol use						0.662
No	114 (95.0)	28 (93.3)	28 (93.4)	30 (100.0)	28 (93.4)	
Irregular	4 (3.3)	2 (6.7)	1 (3.3)	0 (0.0)	1 (3.3)	
Regular	2 (1.7)	0 (0.0)	1 (3.3)	0 (0.0)	1 (3.3)	

Self-perceived health status						0.397
Fair	61 (50.8)	17 (56.7)	12 (40.0)	13 (43.4)	19 (63.3)	
Good or very good	52 (43.3)	11 (36.7)	15 (50.0)	15 (50.0)	11 (36.7)	
Poor or very poor	7 (5.8)	2 (6.6)	3 (10.0)	2 (6.6)	0 (0.0)	

SD: standard deviation.

**Table 2:** Total scores of the State-Trait Anxiety Inventory-State Anxiety Scale (STAI-S) for the control, mandala coloring, plaid pattern drawing, and free-form drawing groups at baseline, postanxiety induction, and postintervention.

Time	Anxiety level, mean (standard deviation)				*P*
	Control	Mandala coloring	Plaid pattern coloring	Free-form color drawing	
T1 (baseline)	34.2 (10.6)	31.1 (7.5)	34.8 (9.8)	35.0 (9.2)	0.346
T2 (postanxiety induction)	35.3 (10.2)	38.0 (11.3)	39.1 (12.0)	40.0 (13.1)	0.457
T3 (postintervention)	34.8 (11.8)	28.3 (6.8)	32.9 (10.2)	30.6 (9.20)	0.060

**Table 3:** Least square-adjusted means of the State-Trait Anxiety Inventory-State Anxiety Scale (STAI-S) for the control, mandala coloring, plaid pattern drawing, and free-form drawing groups at postanxiety induction and postintervention.

Time	Anxiety level, least square-adjusted mean (95% confidence interval)				*P*	Partial eta-squared
	Control	Mandala coloring	Plaid pattern coloring	Free-form color drawing		
T2^1^	35.1 (31.4–38.9)	41.2 (37.0–45.3)	39.8 (35.8–43.8)	40.8 (36.5–45.0)	0.088	0.058
T3^2^	36.0 (32.9–39.2)^a^	28.2 (24.7–31.7)^b^	32.6 (29.2–35.9)^a^	30.2 (26.5–33.8)^a^	0.004	0.113

^1^Value shown is the least square-adjusted mean of STAI-S score adjusting for T1 (baseline) STAI-S score, sex, and educational level. ^2^Value shown is the least square-adjusted mean of STAI-S score adjusting for T2 (postanxiety induction) STAI-S score, sex, and educational level. Least square-adjusted means bearing different superscript letters differ significantly (Šidák post hoc test was used for multiple comparisons, *P* < 0.05).

**Table 4:** Least square-adjusted means of item scores of the State-Trait Anxiety Inventory-State Anxiety Scale (STAI-S) for the control, mandala coloring, plaid pattern drawing, and free-form drawing groups at postintervention.

STAI-S item	Least square-adjusted mean (95% confidence interval)^1^				*P*	Partial eta-squared
	Control	Mandala coloring	Plaid pattern coloring	Free-form color drawing		
Calmed down	2.08 (1.78–2.39)^a^	1.40 (1.05–1.74)^b^	1.83 (1.50–2.16)^a^	1.57 (1.22–1.92)^a^	0.011	0.096
Safe	2.07 (1.75–2.39)^a^	1.35 (1.00–1.70)^b^	2.01 (1.67–2.35)^a^	1.54 (1.18–1.90)^a^	0.002	0.124
Tense	1.54 (1.27–1.80)	1.40 (1.12–1.70)	1.19 (0.91–1.47)	1.20 (0.90–1.50)	0.190	0.042
Annoyed	1.59 (1.33–1.86)	1.40 (1.11–1.69)	1.24 (0.96–1.52)	1.48 (1.17–1.78)	0.285	0.034
Comfortable	2.14 (1.80–2.47)	1.79 (1.42–2.15)	1.88 (1.53–2.24)	1.74 (1.36–2.11)	0.327	0.031
Upset	1.29 (1.12–1.46)	1.08 (0.89–1.27)	1.24 (1.06–1.42)	1.19 (1.00–1.39)	0.317	0.031
Concerned with future misfortunes	1.58 (1.32–1.83)	1.19 (0.91–1.47)	1.24 (0.97–1.51)	1.29 (1.00–1.58)	0.139	0.048
Relaxed	1.96 (1.65–2.27)	1.46 (1.12–1.80)	1.88 (1.55–2.21)	1.65 (1.30–2.01)	0.082	0.059
Anguished	1.16 (0.91–1.40)	1.16 (0.88–1.43)	1.22 (0.96–1.48)	1.08 (0.80–1.37)	0.897	0.005
At ease	1.98 (1.68–2.27)^a^	1.41 (1.08–1.73)^b^	1.86 (1.54–2.18)^a^	1.62 (1.28–1.96)^a^	0.030	0.078
Self-confidence	2.32 (2.03–2.62)	1.82 (1.50–2.14)	2.00 (1.69–2.31)	1.84 (1.50–2.17)	0.057	0.066
Nervous	1.51 (1.24–1.78)	1.30 (1.00–1.60)	1.40 (1.11–1.68)	1.48 (1.18–1.79)	0.675	0.014
Restless	1.82 (1.51–2.13)	1.93 (1.59–2.27)	1.72 (1.40–2.05)	1.96 (1.61–2.32)	0.671	0.014
Downhearted	1.85 (1.58–2.12)	1.63 (1.34–1.93)	1.76 (1.47–2.04)	1.67 (1.37–1.98)	0.652	0.015
Rested	2.18 (1.88–2.47)^a^	1.36 (1.04–1.68)^b^	1.87 (1.56–2.19)^a^	1.76 (1.43–2.10)^a^	0.001	0.130
Satisfied	2.24 (1.96–2.53)^a^	1.28 (0.97–1.60)^b^	1.80 (1.50–2.11)^ab^	1.63 (1.30–1.96)^b^	<0.001	0.176
Concerned	1.38 (1.14–1.62)	1.21 (0.94–1.48)	1.17 (0.91–1.43)	1.13 (0.85–1.40)	0.482	0.022
Stunned	1.44 (1.24–1.64)	1.18 (0.96–1.41)	1.38 (1.17–1.60)	1.33 (1.10–1.56)	0.292	0.033
Happy	1.99 (1.71–2.26)	1.59 (1.29–1.90)	1.94 (1.64–2.24)	1.63 (1.32–1.95)	0.086	0.058
I feel good	1.91 (1.64–2.17)^a^	1.41 (1.12–1.71)^b^	1.91 (1.62–2.19)^a^	1.58 (1.28–1.89)^ab^	0.016	0.089

STAI-S: State-Trait Anxiety Inventory - State Anxiety Scale. ^1^Value shown is the least square-adjusted mean of the corresponding item score of STAI-S score at T3, adjusting for T2 (postanxiety induction) STAI-S score, sex, and educational level. Least square-adjusted means bearing different superscript letters differ significantly (Šidák post hoc test was used for multiple comparisons, *P* < 0.05).

## Data Availability

The data used and analyzed in the present article are available from the corresponding author on reasonable request.
